# Clinical application of 3D printing-assisted patient-specific instrument osteotomy guide in stiff clubfoot: preliminary findings

**DOI:** 10.1186/s13018-023-04341-z

**Published:** 2023-11-07

**Authors:** Wei Liu, Siping Zhang, Wenhao Zhang, Fei Li, Aihelamu Tueraili, Ling Qi, Chengwei Wang

**Affiliations:** 1 The Affiliated Tumor Hospital of Xinjiang Medical University, Ürümqi, 830000 Xinjiang People’s Republic of China; 2 The Sixth Affiliated Hospital of Xinjiang Medical University, Ürümqi, 830000 Xinjiang People’s Republic of China

**Keywords:** Stiff clubfoot, 3D printing, Patient-specific instruments, Surgical, Navigation

## Abstract

**Background:**

The orthopedic treatment of the stiff clubfoot is challenging for clinicians, and the purpose of this study was to explore the preliminary findings of 3D printing-assisted patient-specific instrument (PSI) osteotomy guide for use in the orthopedic treatment of the stiff clubfoot.

**Material and methods:**

There were 20 patients (25 feet) with stiff clubfoot admitted from December 2018 to June 2022, including 13 males (16 feet) and 7 females (9 feet), aged 24–52 years, mean 40.15 years; 8 left feet, 7 right feet, 5 bipedal. All patients underwent triple arthrodesis and were divided into 10 cases (12 feet) in the PSI group (*n* = 12) and 10 cases (13 feet) in the conventional surgery group (*n* = 13) according to the surgical approach. The duration of surgery and the number of radiation exposures were recorded in all cases, and the American Orthopedic Foot and Ankle Society (AOFAS), and International Congenital Clubfoot Study Group (ICFSG) scoring systems were applied postoperatively to assess the effect of corrective treatment. All measurement data were expressed as mean ± standard deviation, and differences between groups were determined by Student’s *t* test. All count data between the two groups were compared using the chi-square test or Fisher’s exact test analysis.

**Results:**

All 20 patients (25 feet) were followed up for 1 year. No major complications related to osteotomy, such as overcorrection, incomplete correction, or bone nonunion, were observed in the PSI and conventional surgery groups at the final follow-up, and the PSI group had the advantage of shorter operative time (*P* < 0.01), less radiation exposure (*P* < 0.01), and higher excellent rate compared with the conventional surgery group. The AOFAS score (*P* > 0.05) and ICFSG score (*P* > 0.05) at the last follow-up were not statistically significant in both groups, but the excellent rate at the last follow-up was 91.7% in the PSI group which was significantly higher than that of the conventional surgery group at 76.9%.

**Conclusions:**

The utilization of 3D printing-assisted PSI osteotomy guide in orthopedic surgery for stiff clubfoot offers a safe and effective surgical tool for triple joint fusion treatment. This technology simplifies surgical procedures, minimizes intraoperative radiation exposures, reduces surgical time, and enables precise and personalized treatment.

## Introduction

Talipes equinovarus (TEV) is a congenital or acquired foot deformity caused by a variety of factors, mainly manifesting as forefoot varus, enlarged longitudinal arch, hindfoot varus and ankle plantar flexion, according to statistics, 1–2 out of every 1000 live births, 80% of which are born in low- and middle-income countries [1, 2]. About half of the patients have unilateral clubfoot deformity [3]. If left untreated or improperly treated, the patient's bones become stiff, misaligned and exposed to abnormal forces, which can exacerbate changes in the anatomical position of the bony structures, and as a result of the use of the misaligned foot to transmit forces during walking, TEV can worsen over time and with growth and developmental changes, gradually evolving into a stiff clubfoot, In addition, severe trauma, burns, interfascial compartment syndrome, peripheral nerve injury and central nervous system injury are also important reasons of normal feet evolving into stiff clubfoot [4].

Triple arthrodesis (TA) is a representative procedure for the correction of Stiff clubfoot [5, 6]. TA refers to the combined fusion of three joints: talocalcaneal, talonavicular, and calcaneocuboid joints, which is mainly applied to patients with severe joint destruction or narrowed joint space. In the case of Stiff clubfoot, a combination of limited osteotomy is required to perform TA to correct the severe three-dimensional deformity of clubfoot, and the operator's extensive experience, effective preoperative evaluation and design of the preoperative surgical plan are critical factors affecting the final outcome of TA. With the increase in the number of TA cases in Stiff clubfoot and the continuous development of the technique, the postoperative complications associated with TA are gradually becoming known to the physicians. Overcorrection, incomplete correction, residual symptoms in the affected foot, and postoperative degenerative changes in the adjacent joints are all common complications after TA correction of Stiff clubfoot [7, 8]. How to avoid or reduce the occurrence of postoperative complications and improve the excellent rate of surgery is the goal pursued by physicians in recent years. Traditional TA surgery is to determine the degree of deformity of the affected limb and the angle of osteotomy correction, osteotomy volume and osteotomy method through the subjective judgment of the attending surgeon on the digital image of the patient, which lacks objective basis [9]. Even experienced surgeons encounter challenges when it comes to making precise osteotomies during surgery. They often rely on a combination of constant osteotomies and fluoroscopy to determine the final osteotomy angle. While this approach enhances the precision of surgical osteotomies, it also leads to increased operating time, intraoperative radiation exposure, and a higher risk of postoperative complications [10]. Given the adverse consequences of complications following Stiff clubfoot orthopedic surgery, more objective, precise and individualized means are needed to advance TA in the treatment of Stiff clubfoot。

In recent years, 3D printing technology has been widely used in the field of orthopedics, which can quickly build accurate and complete skeletal models, making it easier for orthopedic surgeons to observe, measure and even practice simulated surgery on the models more intuitively [11]. It is also possible to construct 3D printing-assisted PSI osteotomy guide to accurately and conveniently assist complex osteotomy and repositioning operations in orthopedic surgery, thereby improving the efficiency of osteotomy and repositioning. Therefore, to further evaluate the safety and efficacy of PSI in stiff clubfoot TA, this study retrospectively compared the results of PSI-assisted surgery with conventional surgery and evaluated the prognosis.

## Materials and methods

### Patients

Twenty (25 foot) (≥ 18 years old) stiff clubfoot patients treated with TA at the Sixth Affiliated Hospital of Xinjiang Medical University from December 2018 to May 2022 were included in this retrospective study, including 13 (16 foot) males and 7 (9 foot) females, aged 24–52 years, mean 40.15 years; 8 left foot, 7 right foot, and 5 bipedal patients. Inclusion and exclusion criteria were used (see Table 1). Those included were randomly divided into 10 cases (12 feet) in the PSI group (*n* = 12) and 10 cases (13 feet) in the conventional surgery group (*n* = 13), with no statistically significant differences in age, gender, affected side, AOFAS score, and ICFSG score between the two groups (see Table 2). TA was performed in both groups, and all procedures were performed by the same team. The study was approved by the ethical review committee of the Sixth Affiliated Hospital of Xinjiang Medical University, and all participants signed an informed consent form.

**Table 1:** The inclusion and exclusion criteria

The inclusion criteria	The exclusion criteria
Type III (severe) or higher according to the Diméglio classification	Local soft tissue conditions do not meet the criteria for surgical treatment
Previous conservative treatment is ineffective or has poor results	Patients aged < 18 years
No other soft tissue, joint or bone lesions except for clubfoot deformity	Other types of clubfoot, such as postural, syndromic, and combined with other deformities

**Table 2:** Comparison of demographic data and deformity characteristics between two groups

Characteristics	Navigation template group (*n* = 12)	Conventional group (*n* = 13)	*P* value
Mean age (range), years	39.42 ± 6.79 (24–49)	42.31 ± 7.41 (26–52)	0.321
Gender, *n* (%)			0.790
Male	8 (66.7)	8 (61.5)	
Female	4 (33.3)	5 (38.5)	
Stiff clubfoot malformation side, n (%)			0.848
Left	6 (50)	7 (53.8)	
Right	6 (50)	6 (46.2)	
AOFAS score, point	45.17 ± 2.12	44.31 ± 3.22	0.444
ICFSG score, *n* (%)			
Excellent	0	0	–
Good	0	0	–
Fair	0	0	–
Poor	12 (100)	13 (100)	0.671

Data are presented as mean ± standard deviation or counts (percentages). Differences were considered statistically significant at *P* < 0.05

*n* number of cases; *AOFAS* American Orthopedic Foot and Ankle Society; *ICFSG* International Congenital Clubfoot Study Group

### Digital design and PSI preparation

**Fig. 1:**
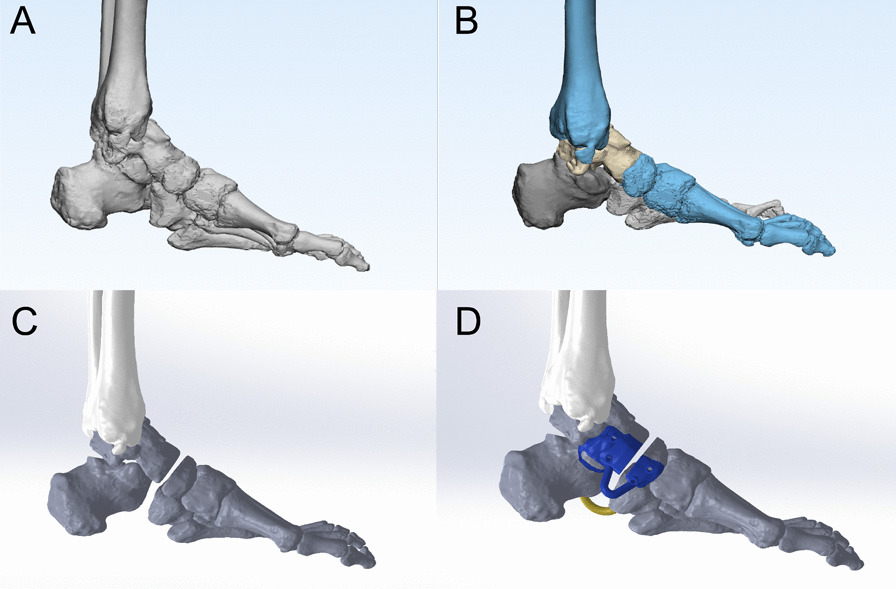
Design of PSI osteotomy guide plate. **a** 3D skeletal model of the affected limb. **b** Adjustment of the skeletal position of the foot to simulate the skeletal position relationship after orthopedic surgery. **c** The overlapping part is used as the surgical excision part, and then, the model position is recovered. **d** Building a PSI osteotomy guide plate model according to the surgical osteotomy protocol

All patients were scanned with X-ray and dual-source 64-row spiral CT (Siemens, Germany) with the following parameters: voltage 120 kV, current intensity 240 mA, and slice thickness 0.60 mm. The CT scan data of the PSI group were imported into Mimics 21.0 software (Materialise, Belgium) in digital imaging and communications in medicine (DICOM) format, and the 3D model was reconstructed as a STereoLithography (STL) file. The foot skeleton model in STL format was imported into Geomagic Wrap software (Raindrop, USA), and the non-uniform Rational B-Splines (NURBS) surface model of the foot skeleton was obtained by removing pegs, noise reduction, smoothing, and fine surface operations, and the obtained model was imported into SolidWorks software (Dassault, France). SolidWorks software (Dassault, France) was used to adjust the position of the foot bones, simulate the position of the bones after orthopedic surgery, record the parameters of each adjustment step, mark the overlapping position of the bones, use the overlapping part as the surgical resection part, recover the model position, use the size of the field of view of the operating area as the base design range of the navigation template, determine the plane, angle and depth of osteotomy based on the marking position, and perform the simulation after osteotomy. The osteotomy end is reset, and the corrected shape is measured again to check whether the deformity is corrected. The size of the PSI was reduced as much as possible within the field of view of the operative area to ensure accuracy and strength, and to minimize the incision. Due to the interference of factors such as periosteum and soft tissue obstruction on the bone, a 2-mm interval was set between the PSI and the bone. In order to increase the stability of the PSI during surgery, two large holes of 3 mm diameter were designed on the PSI as the fixation holes for the kerf pins. In order to ensure the accuracy of the PSI, the fitting surface of the guide plate was aligned with the bone surface. The printing of the foot bone and PSI model was completed by 3D printing technology, and the preoperative surgical simulation was completed, and the perfect fitting of the bone and PSI could be seen in the 3D printed model, and the height of the guide blade slot was set to about 5 mm, which could ensure the PSI had a good guiding effect (Fig. 1).

### Surgical procedures

**Fig. 2:**
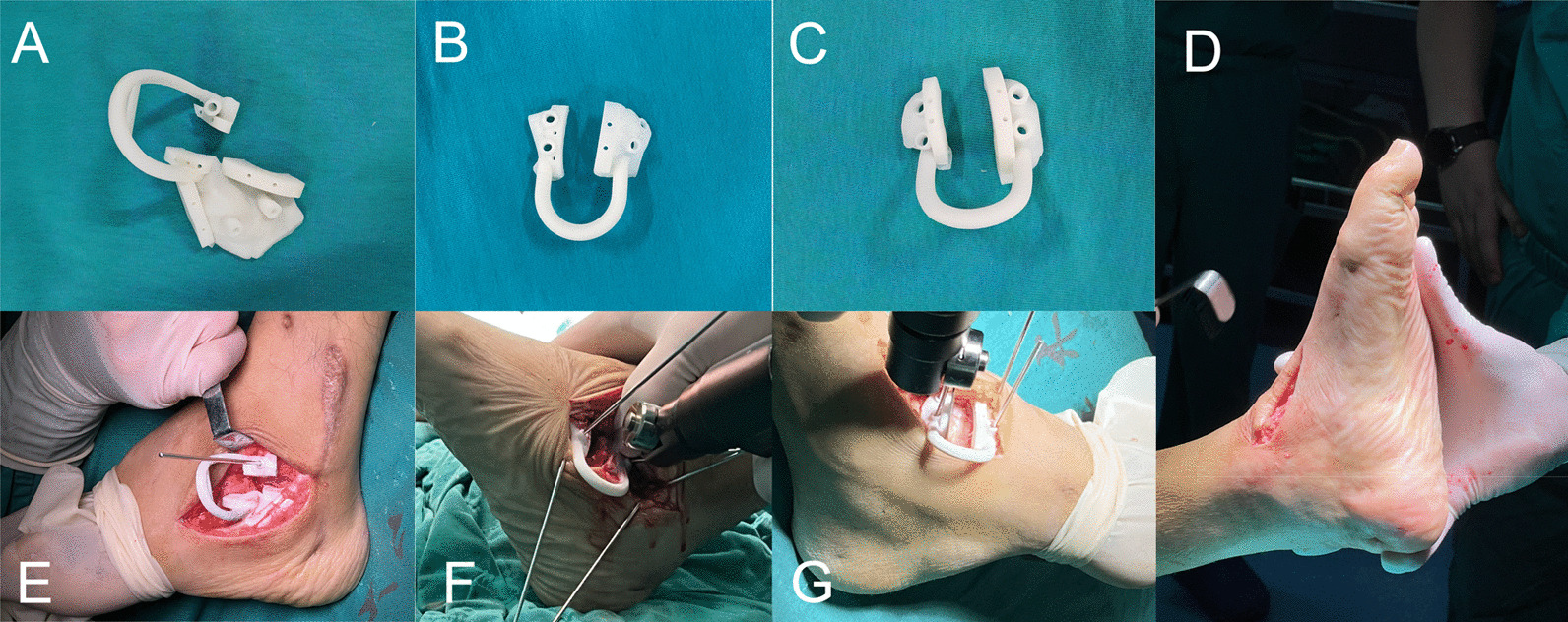
Intraoperative application of PSI. **a** Subtalar joint template. **b** Calcaneocuboid joint template. **c** Talonavicular joint template. **d** Intraoperative observation of orthopedic results. **e** Intraoperative application of subtalar joint template. **f** Intraoperative application of calcaneocuboid joint template. **g** Intraoperative application of talonavicular joint template

The anesthesia method was chosen as epidural anesthesia, and after anesthesia took effect, the patient was placed in a flat position, the proximal thigh of the affected side was tied with a balloon blood repellent band, and a routine disinfection of the towel was laid, and the affected limb was controlled with a balloon blood repellent band after the blood was repelled. In the PSI group, a transverse incision of approximately 5 cm in length was made below the tarsal sinus of the external ankle, while paying attention to the protection of neurovascularity, the skin and subcutaneous tissue were incised layer by layer to reveal the talocalcaneal joint, a sterile talocalcaneal joint PSI was taken and placed in the incision, and after complete fitting, four 2-mm Kirschner nails were placed in the surrounding positioning holes according to the preoperative simulation. The positioning and fixation of the joint are performed, after seeing that the talocalcaneal joint PSI is well-fitted and stable, the pendulum saw is aligned with the guiding hole of the talocalcaneal joint PSI, and a wedge-shaped osteotomy is performed according to the osteotomy depth obtained from the preoperative simulation, and after the osteotomy is completed, the talocalcaneal joint PSI is removed, after manual repositioning, the talocalcaneal joint is temporarily fixed using a Kirschner nails, C-arm fluoroscopy shows good repositioning, and all-thread hollow nail fixation is taken. Then, a transverse incision of about 4 cm in length was made medial to the talonavicular joint to reveal the talonavicular joint, and a sterile talonavicular joint PSI was taken and placed in the incision, after complete fitting, four 2-mm Kirschner nails were inserted into the positioning holes according to the preoperative simulation to provide positioning and fixation. After seeing that the talonavicular joint PSI is well-fitted and stable, the pendulum saw is aligned with the guiding hole of the talonavicular joint PSI and a wedge-shaped osteotomy is performed according to the osteotomy depth obtained from the preoperative simulation. After the osteotomy is completed, the sterile talonavicular joint PSI is taken, manually repositioned, fixed with Kirschner nails, intraoperative fluoroscopic repositioning is possible, and fixed with portal nails. A 2-cm incision was made at the lateral calcaneocuboid joint of the foot, and the lateral column was shortened and fused to the calcaneocuboid joint according to the preoperative simulated thickness, placement of portal nails, fused calcaneocuboid joint. The ankle joint internal and external rotation, dorsiflexion and plantarflexion, forefoot shape correction, hindfoot force line positive (Fig. 2), a large amount of saline rewash the wound surface to avoid foreign body residue, affecting the incision and bone healing, layer by layer suture. After inventorying the intraoperative objects, the affected limb was bandaged and external fixation in plaster was performed. In contrast, the determination of the osteotomy line for the traditional surgical group is based primarily on preoperative planning and the number of intraoperative attempts. The main procedures include correction of forefoot and hindfoot varus deformities and midfoot inversion deformities, after confirming that the osteotomy is satisfactory, the talocalcaneal joint is fixed with hollow screws, fixation of the talonavicular and calcaneocuboid joints with a portal nail.

### Statistical analysis

Data presented in this current research were statistically analyzed by the SPSS 26.0 software (SPSS, USA) and exhibited as mean ± standard deviation (SD) or count (percentage). The statistical methods of Chi-squared test, Fisher exact test, and Student’s t test were applied in this research. The independent t test was used to assess the continuous variables, and the chi-square test or Fisher exact test was used to assess the categorical variables of different parameters collected between the two groups. *P* value < 0.05 was represented as statistically significant.

## Results

**Fig. 3:**
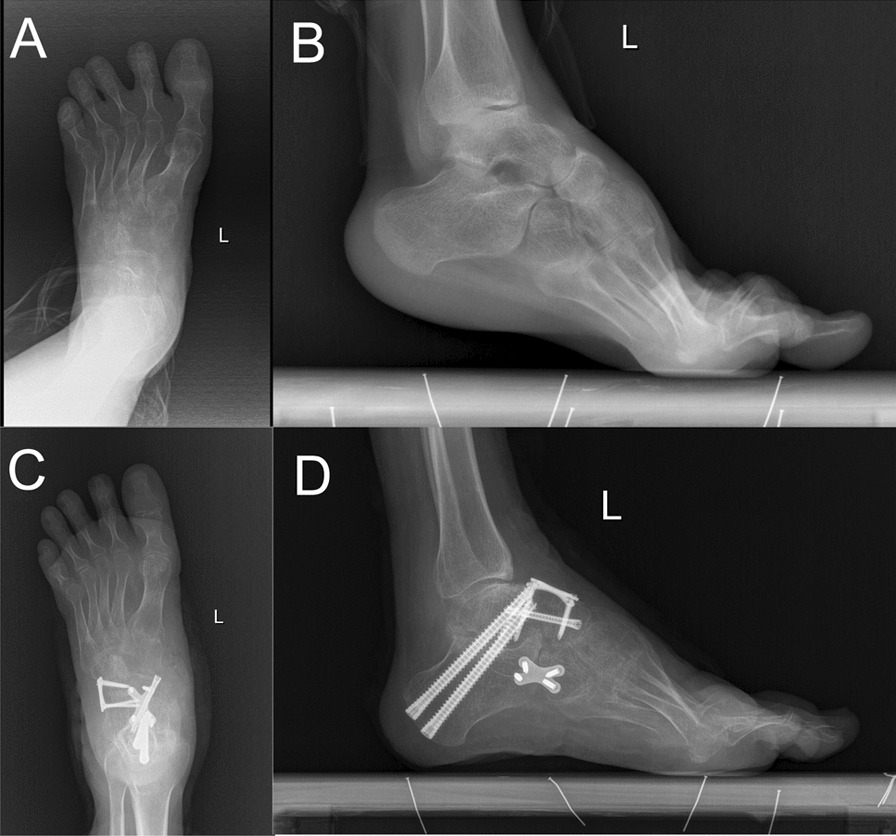
Results of pre- and post-operative X-rays. **a** Preoperative anterior–posterior foot X-ray. **b** Preoperative lateral foot X-ray. **c** Postoperative anterior–posterior foot X-ray. **d** Postoperative lateral foot X-ray

A total of 20 (25 foot) stiff clubfoot patients were included in this study, 10 (12 foot) in the PSI group and 10 (13 foot) in the conventional surgery group. There was no statistically significant difference between the two groups in terms of preoperative age, sex, number of deformed sides, AOFAS score, and ICFSG score (*P* > 0.05), and both groups were followed up for a period of 1 year. The duration of surgery was 60.67 ± 6.15 min in the PSI group compared to 78.23 ± 7.50 min in the conventional surgery group (*P* < 0.01), and the number of radiation exposures was 3.58 ± 0.67 in the PSI group compared to 6.69 ± 1.25 in the conventional surgery group (*P* < 0.01), and the differences were statistically significant. At the final follow-up, there were no osteotomy-related complications such as overcorrection, incomplete correction, or bone non-union in both PSI and conventional surgery groups, and satisfactory results were obtained in both groups (Fig. 3). The difference between the AOFAS score of 77.58 ± 2.35 at the last follow-up in the PSI group and the AOFAS score of 76.92 ± 2.25 at the last follow-up in the conventional surgery group was not statistically significant (*P* > 0.05), and the difference between the ICFSG score at the last follow-up in the PSI group and the ICFSG score at the last follow-up in the conventional surgery group was not statistically significant, but the superiority rate of 91.7% was significantly higher than that of 76.9% in the conventional surgery group (see Table 3).

**Table 3:** Comparison of operation data and functional outcomes between two groups

	Navigation template group (*n* = 12)	Conventional group (*n* = 13)	*P* value
Operative time, min	60.67 ± 6.15	78.23 ± 7.50	< 0.01
X-ray exposure, times	3.58 ± 0.67	6.69 ± 1.25	< 0.01
AOFAS score at last follow-up, point	77.58 ± 2.35	76.92 ± 2.25	0.481
ICFSG score at last follow-up, *n* (%)			
Excellent	2(16.7)	1(7.7)	1
Good	9(75.0)	9(69.2)	0.790
Fair	1(8.3)	3(23.1)	0.478
Poor	0	0	–
Rate of excellent and good outcomes, %	91.7	76.9	0.270

Data are presented as mean ± standard deviation or counts (percentages). Differences were considered statistically significant at *P* < 0.05

*n* number of cases; *AOFAS* American Orthopedic Foot and Ankle Society; *ICFSG* International Congenital Clubfoot Study Group

## Discussion

The stiff clubfoot is a complex three-dimensional deformity, and the goal of surgical treatment is to obtain a painless, deformity-free appearance of the metatarsal foot [12], safe and precise orthopedic surgery is the key to accomplishing the goal. In the past, precise control of all dimensions of orthopedic treatment was difficult [13], and the surgical procedure often requires a series of operations such as repeated osteotomy adjustment, temporary fixation, X-ray fluoroscopy, and observation of the foot shape to determine the degree of correction, which undoubtedly increases the risk of overcorrection or incomplete correction [14]. With the increasing demand for individualized medicine and the widespread use of 3D printing technology in orthopedics, PSI has been accepted and used by most orthopedic surgeons for its simplicity, practicality and precision [15, 16], and achieved satisfactory results [17]. Therefore, in this study, we applied 3D digital technology and 3D printing to complete the orthopedic surgery of stiff clubfoot and compared it with the traditional surgical approach to evaluate the safety and the effectiveness of 3D printing-assisted PSI osteotomy guide in the application of stiff clubfoot orthopedic surgery.

We retrospectively compared the efficacy evaluation of conventional surgery and PSI-assisted correction of stiff clubfoot deformity. The PSI designed in this study is simple, small in size, reduces the surgical incision, easy to operate, the angle between the osteotomy surfaces on both sides of the guide slot is the angle of deformity correction, and the navigation template is fixed by four kerf pins position on the PSI to avoid errors caused by intraoperative displacement. In the PSI group, the individualized 3D-printed navigation template was implemented with the same preoperative planning, the guide was tightly fitted to the operative area and firmly fixed, and all patients were successfully osteotomized in a single pass, avoiding repeated osteotomy adjustments and X-ray fluoroscopy and simplifying the surgical procedure, which explained the difference in operative time in the PSI group (60.67 ± 6.15 vs. 78.23 ± 7.50, *P* < 0.01) and number of radiation exposures (3.58 ± 0.67 vs. 6.69 ± 1.25, *P* < 0.01) were significantly less than those of the conventional surgery group. Similarly, the findings of other scholars are more consistent with ours, and PSI has been widely used in the field of orthopedics [18–20]. Zheng et al. [21] applied PSI to 12 older (10.85 ± 2.02 years old) patients with congenital hip dysplasia, and compared with 13 patients who were close at baseline and underwent the same surgery but did not use PSI, their operative time, number of X-ray exposures, and incidence of epiphyseal injuries were significantly reduced, as shown by the follow-up results after 12–18 months postoperatively. However, there was no statistically significant difference in the follow-up results between the two groups, and it was concluded that the application of PSI to older children with congenital hip dysplasia could reduce operative time, X-ray exposure, and epiphyseal injury, as well as simplify the procedure and improve accuracy, Zhang et al. [22] similarly designed a PSI and applied it to stiff clubfoot patients; a total of 27 patients were included, 15 of whom had the PSI applied and 12 underwent conventional surgery with midfoot osteotomy fusion. The results of the study showed that compared with the conventional group, the PSI group had significant advantages in terms of shorter operative time, less intraoperative bleeding, higher excellent rate, and ICFSG score performance at the last follow-up, but the large amount of osteotomy and the inevitable postoperative shortening of the foot length seriously affected the overall appearance of the foot, therefore, this project used TA to correct the deformity [23], a greater degree of foot shortening is avoided, preserving the overall beauty of the foot [24]. This is the key to improving patient satisfaction.

Furthermore, in this study, satisfactory results were obtained in both groups from the end follow-up, indicating that TA is still an effective surgical procedure for the correction of stiff clubfoot. Peterson g et al. [25] concluded that clubfoot is a bi-directional problem in time and space, with time changing the nature and ultimate prognosis of the deformity and the spatial structure changing the function of the foot. As patients age and lose the potential for skeletal remodeling of the foot, the only way to change the shape of the foot and thus restore foot function is through osteotomy and fusion surgery. This is why TA has become an important reason why stiff clubfoot orthopedics is the most popular surgical procedure. The differences in AOFAS score (77.58 ± 2.35 vs. 76.92 ± 2.25, *P* > 0.01) and ICFSG score in the PSI group were not statistically significant compared with the conventional surgery group, but the excellent rate in the PSI group (91.7% vs. 76.9%) was significantly higher than that in the conventional surgery group; therefore, we believe that the PSI-assisted correction of stiff clubfoot deformity can provide precise and personalized treatment with better stability and safety for stiff clubfoot patients.

This study also has some limitations: First, the number of cases in which the osteotomy was performed using the navigation template was small, the follow-up time was insufficient, and the results were not compared with those of the conventional surgery group over time. Future studies will focus on larger samples and long-term follow-up studies with the application of this PSI. Second, although this study shortened the surgical time and increased the surgical precision for each volunteer, the time period spent on the processing of CT data, the design of the surgical plan, and the design of the PSI was long, and the time period required for preoperative planning was significantly longer than that required for traditional direct film review, and the entire preoperative planning required learning and mastering the relevant digital software, which significantly increased the time cost. Third, the specific comparison of preoperative and postoperative lower extremity imaging alignment parameters between the two study groups is also significant and will be further refined in future studies.

## Conclusion

The application of 3D printing-assisted PSI osteotomy guide for the correction of stiff clubfoot is safe, precise, and accurate. Compared with the conventional group, the PSI group has the advantages of shorter operative time and less radiation exposure. Stiff clubfoot treatment with PSI is feasible and effective, which provides a safe and effective surgical tool for TA treatment of stiff clubfoot.

## Data Availability

The datasets used and/or analyzed during the current study are available from the corresponding author on reasonable request.

## Abbreviations

PSI: Patient-specific instrument
AOFAS: American Orthopedic Foot and Ankle Society
ICFSG: International Congenital Clubfoot Study Group
TA: Triple arthrodesis
TEV: Talipes equinovarus
CT: Computed tomography
DICOM: Digital imaging and communications in medicine
NURBS: Nonuniform rational B-splines
